# Low-Frequency rTMS of the Primary Motor Area Does Not Modify the Response of the Cerebral Cortex to Phasic Nociceptive Stimuli

**DOI:** 10.3389/fnins.2018.00878

**Published:** 2018-11-29

**Authors:** Costanza Pazzaglia, Catello Vollono, Elisa Testani, Daniele Coraci, Giuseppe Granata, Luca Padua, Massimiliano Valeriani

**Affiliations:** ^1^Unit of High Intensity Neurorehabilitation, Fondazione Policlinico Universitario A. Gemelli, IRCCS, Rome, Italy; ^2^Department of Geriatrics, Neuroscience and Orthopedics, Fondazione Policlinico Universitario A. Gemelli, IRCCS, Rome, Italy; ^3^Department of Neurological and Neurosensory Sciences, University Hospital of Siena, Siena, Italy; ^4^IRCCS Fondazione Don Carlo Gnocchi, Milan, Italy; ^5^Department of Geriatrics, Neurosciences and Orthopedics, Catholic University of the Sacred Heart, Rome, Italy; ^6^Department of Neuroscience, Pediatric Hospital Bambino Gesù, Rome, Italy; ^7^Center for Sensory-Motor Interaction, Aalborg University, Aalborg, Denmark

**Keywords:** rTMS, low-frequency, motor cortex, laser evoked potential, pain processing

## Abstract

Repetitive transcranial magnetic stimulation (rTMS) is a non-invasive technique of cortical stimulation. Although the exact mechanism of action is not clearly understood, it has been postulated that rTMS action on pain depends most on stimulation sites and stimulation parameters. Most studies concern high-frequency rTMS of the primary motor cortex (M1). High-frequency rTMS over motor cortex seems to induce an analgesic effect while contrasting results were reported after low-frequency rTMS. The aim of the current study was to investigate the effects of 1 Hz rTMS stimulation over the left primary motor cortex on subjective laser pain rating and laser evoked potential (LEP) amplitudes in healthy subjects. Subjects underwent two different sessions (real and sham rTMS) according to a cross-sectional design. In each session, LEPs and laser-pain rating to stimulation of both right and left hand dorsum were collected before 1 Hz rTMS over the left M1 area (baseline), which lasted 20 min. Then, LEPs and laser-pain rating were measured immediately after rTMS (T0), after 20 min from T0 (T0+20), and after 40 min from T0 (T0+40). We could not find any modification of both laser-pain rating and LEP parameters (latencies and amplitudes) following 1 Hz rTMS. Therefore, our results show that the low-frequency rTMS of the M1 area does not change the response of the cerebral cortex to pain.

## Introduction

Repetitive transcranial magnetic stimulation (rTMS) is a non-invasive technique of cerebral cortex stimulation. The magnetic field delivered by a coil determines electric currents in neurons able to modify the excitability of neuronal networks in the cortex (Fregni and Pascual-Leone, 2007; Leo and Latif, 2007). The magnitude of actions relies on coil type and orientation, magnetic pulse waveform, stimulation pattern, distance between the coil and the cerebral areas, and stimulated area (Lefaucheur et al., 2014).

Repetitive transcranial magnetic stimulation specific action on pain seems to be related to several pain modulatory systems such as, endogenous opioids, gabaergic circuitry, dopamine, and serotonine modification (Moisset et al., 2016). Also modifications of the cerebral blood flow in the pain matrix areas and effects on the emotional brain centers could contribute to the rTMS effect (Tamura et al., 2004b). Although mechanisms of action have not been clearly understood, rTMS action on pain depends on the stimulation site and parameters, such as duration and frequency of stimuli (Moisset et al., 2016). Primary motor cortex (M1) and dorsolateral prefrontal cortex (DLPFC) have been more often stimulated up to now (Brighina et al., 2011; Short et al., 2011; Hosomi et al., 2013; Moisset et al., 2015). These cortical regions interact with those specifically devoted to pain processing and with associative cortex elaborating the attentive and emotional compound of relevant stimuli. Since nociception is not only due to the activation of certain “pain areas,” while it probably depends on the parallel activation of sensory, motor and limbic areas (Garcia-Larrea and Bastuji, 2018), the concept of “pain matrix” should be addressed as “nociceptive matrix.”

As general rule, low-frequency rTMS (≤1 Hz) results in lowered cortical excitability at the site of stimulation (Fregni et al., 2011; Sampson et al., 2011), whereas high-frequency stimulation (≥5 Hz) leads to raised cortical excitability (Pascual-Leone et al., 1998; Lefaucheur, 2008). While there is a certain agreement in showing that high-frequency stimulation of both M1 area (Lefaucheur et al., 2001; André-Obadia et al., 2006) and DLPFC provides an analgesic action (Borckardt et al., 2009; Ciampi de Andrade et al., 2014), few and contrasting results have been found with the low-frequency rTMS (Tamura et al., 2004b; André-Obadia et al., 2006). Although the precise mechanism underlying analgesia due to high-frequency stimulation of the M1 area is not understood, it can be supposed that high-frequency rTMS may potentiate the inhibitory influence of the M1 area on the nociceptive cortex (Le Pera et al., 2007). Seen in this light, the inhibition of the M1 area, obtained by low-frequency rTMS, should facilitate pain perception. In painful conditions, the unilateral stimulation of M1 and DLPFC determines a bilateral and selective effect independently of the stimulated side, this was demonstrated both in healthy and patients affected by fibromyalgia, respectively (Passard et al., 2007; Nahmias et al., 2009). In both studies the unilateral stimulation was able not only to induce a diffuse but also a selective effect on different pain modalities. This is different from what happens in other disease, e.g., Parkinson disease, where the stimulation of left striatum determines an ipsilateral release of dopamine (Cho and Strafella, 2009; Chervyakov et al., 2015), thus allowing the possible identification of neurobiological action of TMS for the treatment of several neurological conditions.

Indeed less is known about the action of low-frequency rTMS, while Tamura et al. (2004a) showed that 1 Hz rTMS of the M1 area increased pain perception and the amplitude of the pain-evoked brain responses, this result has not been confirmed by other studies (André-Obadia et al., 2006; Saitoh et al., 2007; Lefaucheur et al., 2008).

Laser evoked potentials (LEPs) are considered the most reliable tool to assess the function of nociceptive pathways in humans (Valeriani et al., 2012). Specifically, laser pulses are able to activate the thin myelinated (Aδ) and unmyelinated (C) fibers selectively, without any stimulation of the Aβ afferents. The main cerebral responses evoked by laser stimulation of the skin are represented by the N1 component, identifiable in the contralateral temporal region and probably generated in the opercular (SII/insula) area, and the N2/P2 complex, widely diffused over the scalp and mostly originated from the anterior cingulate cortex (Garcia-Larrea et al., 2003).

In healthy subjects, the LEP amplitude can be considered as an objective measure of pain processing that can be influenced by external conditioning stimuli.

The aim of the current study was to investigate the effects of 1 Hz rTMS stimulation over the left primary motor cortex on subjective laser pain rating and LEP amplitudes in healthy subjects. We hypothesized that both parameters should be increased by the M1 area inhibition.

## Materials and Methods

Ten healthy right handed volunteers were enrolled (5 males, 5 females, mean age: 26.5 ± 12.4 years). Subjects were excluded in case of symptoms or signs of focal upper limb entrapment, cervicobrachialgia or polyneuropathy. Also subjects affected by painful conditions were excluded.

This study was carried out in accordance with the recommendation of Don Carlo Gnocchi Foundation local Ethics Committee. The protocol was approved by the Don Carlo Gnocchi Foundation local Ethics Committee. All subjects gave written informed consent in accordance with declaration of Helsinki. Subjects can withdraw from the study at any time.

**FIGURE 1:**
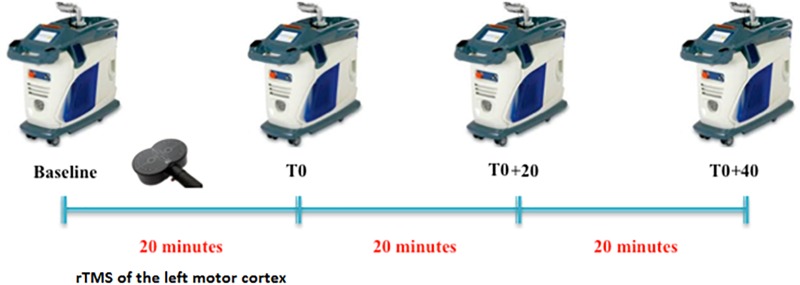
Experimental protocol. For each condition (baseline, T0, T0+20, and T0+40), LEPs were recorded to both right and left hand stimulation.

The subjects were evaluated in two different sessions (real and sham rTMS) according to a cross-sectional design. In each session, LEPs were recorded to stimulation of both right and left hand dorsum in a baseline condition (before rTMS) and in three stimulation conditions: (1) immediately after 20 min of 1 Hz rTMS of the hand motor area of the left hemisphere (T0), (2) after 20 min from T0 (T0+20), and after 40 min from T0 (T0+40) (Figure 1). The order of the sessions was randomized across all subjects. All session were performed in the afternoon and real and sham sessions were separated by an interval ranging from 7 to 14 days.

### Laser Stimulation and LEP Recording

Laser pulses (wavelength, 1.34 μm) were delivered by a YAP Stimul 1340 (Electronic Engineering, Florence, Italy). Laser stimulus intensity was fixed at 38 mJ/mm2 (slightly over the pain threshold), perceived by participants as a painful pinprick (Valeriani et al., 2002; Cruccu et al., 2003). The interstimulus interval varied randomly between 9 and 11 s.

Laser evoked potentials were recorded from two midline electrodes (Fz and Cz positions of the 10–20 International System), and one lead in the temporal region contralateral to the stimulation (T3/T4). An electrode placed over the wing of the nose was the reference, while a further electrode on the forehead (Fpz) was the ground. An electrooculographic (EOG) electrode, placed on the right eyebrow, was used to monitor eye movements and eye-blinks. The analysis time was 1000 ms with a bin-width of 2 ms. The filter bandpass ranged from 0.3 to 70 Hz. For each experimental condition and stimulation site, we recorded averages of 25–30 trials. Since attention can be diverged from the painful stimulation during the experiment, subjects were asked to count the number of the received laser stimuli silently. Averages with a percentage of mistakes higher than 10% were discarded. The subjective laser-pain intensity was rated by a 100 mm visual analog scale (VAS), ranging from “0” (no pain) to “100” (the worst imaginable pain). VAS was administered after each LEP recording.

### Low-Frequency rTMS

Repetitive transcranial magnetic stimulation was applied on left motor cortex by using MagVenture equipment [Cool-B65 figure-of-eight coil (F8), coupled to a MagProX100 stimulator]. All our subjects were right handed. We decided to stimulate only the left motor cortex, since previous studies reported that the unilateral stimulation determines a diffuse analgesic effect independently on the stimulated side (Passard et al., 2007; Nahmias et al., 2009).

Sham stimulation was performed by orientating the coil perpendicular to the scalp. For the real stimulation, the coil handle was pointed backwards at 45° from the midline and biphasic pulses (280 μs) were delivered. First, we localized the first dorsal interosseous muscle motor hotspot, defined as the point where a fixed-intensity TMS evoked the highest motor evoked potential (MEP) amplitude. Then, the resting motor threshold (RMT) was obtained at a stimulus intensity eliciting MEPs above 50 μV in 5/10 consecutive trials (Rossini et al., 1994). A total of 1200 stimuli were delivered over the hotspot at a frequency of 1 Hz, at 90% RMT in both the real and sham rTMS session.

As it is known the importance and the possible impact of the participant’s expectation respect to the protocol, our subjects received neutral instructions as follows: “you will take part of two different recording sections in which we will study the effects of minimal variation of rTMS protocol on brain function” (Colloca et al., 2004). At the end of the second session, each subjects was required whether he/she had noticed difference between active and sham rTMS protocols and none of them had the impression of a “not working” stimulation technique.

### LEP Analysis and Statistics

Laser evoked potential peak latencies were measured on the contralateral temporal trace for the N1 component and on the Cz trace for both the N2 and P2 potentials. The peak-to-peak N1 amplitude was measured in the trace calculated off line by referring the contralateral temporal electrode to Fz. This procedure makes it easier the N1 labeling, which in the temporal trace referred to the nose can be hampered by muscular noise (Kunde and Treede, 1993). Latency and amplitude values are reported as mean ± 1 standard deviation. VAS values are expressed as mean and interquartile range (IQR).

First of all, the data sets were submitted to Shapiro–Wilk test for normality. Since all of them overtook the test, the statistical analysis was based on two-way ANOVAs. Then, in order to test whether rTMS changed laser pain perception and/or LEP parameters and any possible change was different between real and sham session, VAS values and LEP latencies and amplitudes underwent two-way ANOVAs, by considering the session and the time (baseline, T0, T0+20, and T0+40) as variables. If statistical significance was reached, *post hoc* analysis was conducted by paired Student’s *t*-test. Statistical significance was fixed at 0.05.

## Results

All our subjects showed a middle-latency N1 potential in the contralateral temporal trace. The biphasic negative-positive (N2/P2) response was consistently recorded by the Cz electrode (Table 1).

**Table 1:** Visual analog scale (VAS) and LEP values.

Real rTMS									
		**Baseline**		**T0**		**T0+20**		**T0+40**	

*VAS*	Right hand	43.9 (IQR 16)		41.4 (IQR 30)		48.9 (IQR 22)		50.6 (IQR27)	
	Left hand	42.1 (IQR 7)		48.4 (IQR 20)		53 (IQR 27)		51.1 (IQR 28)	

***LEP values***		**Latency (ms)**	**Amplitude (μV)**	**Latency (ms)**	**Amplitude (μV)**	**Latency (ms)**	**Amplitude (μV)**	**Latency (ms)**	**Amplitude (μV)**

N1	Right hand	156.9 ± 25	5.2 ± 2.8	150.1 ± 27.6	4.8 ± 3	151 ± 25.1	4.8 ± 4	153.6 ± 23	5.1 ± 3.6
	Left hand	159.7 ± 27.2	3.1 ± 1.2	155.2 ± 14.1	4.5 ± 3.1	154.3 ± 18.6	3.6 ± 1.8	149.4 ± 22.4	3 ± 1.8
N2	Right hand	185.4 ± 18.6	–	185.5 ± 20.7	–	179.5 ± 17.4	–	179.8 ± 19.8	–
	Left hand	187.8 ± 25.1	–	185 ± 16	–	190.5 ± 22.4	–	180.3 ± 22.8	–
P2	Right hand	290.1 ± 28.8	–	289 ± 35	–	275.6 ± 33.4	–	271.3 ± 31.7	–
	Left hand	298.9 ± 28	–	280.8 ± 29.4	–	287.1 ± 21.8	–	280.6 ± 25.1	–
N2/P2	Right hand	–	35.6 ± 11.8	–	33.9 ± 11.6	–	29.8 ± 9.5	–	27.4 ± 8.2
	Left hand	–	26.9 ± 11.1	–	29.1 ± 5.8	–	25.1 ± 4.2	–	23.8 ± 5.3

**Sham rTMS**									

		**Baseline**		**T0**		**T0+20**		**T0+40**	

*VAS*	Right hand	49.7 (IQR 26)		49.7 (IQR 27)		51 (IQR 24)		54.6 (IQR 19)	
	Left hand	46.4 (IQR 13)		53.6 (IQR 17)		52.3 (IQR 13)		51.4 (IQR 29)	

***LEP values***		**Latency (ms)**	**Amplitude (μV)**	**Latency (ms)**	**Amplitude (μV)**	**Latency (ms)**	**Amplitude (μV)**	**Latency (ms)**	**Amplitude (μV)**

N1	Right hand	156.2 ± 22.3	6.8 ± 2.1	166.2 ± 24	7.6 ± 5	159.7 ± 24.7	7 ± 4.2	148.1 ± 14.9	4.3 ± 2.1
	Left hand	158.3 ± 28	6.7 ± 3.3	150.7 ± 26	4.3 ± 3.4	153.3 ± 24.6	4.3 ± 2.1	162.2 ± 28.3	3 ± 3
N2	Right hand	185.1 ± 19.3	–	185.5 ± 16.5	–	183.1 ± 16.5	–	183.5 ± 20.6	–
	Left hand	186.2 ± 19.1	–	182.5 ± 18.5	–	187 ± 16.4	–	183.5 ± 20.8	–
P2	Right hand	284.5 ± 18.1	–	287.4 ± 19.9	–	283.4 ± 20.1	–	260.5 ± 25.8	–
	Left hand	281.9 ± 16.9	–	293.9 ± 26.9	–	276.8 ± 38.4	–	273.9 ± 34.9	–
N2/P2	Right hand	–	36.5 ± 15	–	30.7 ± 11.9	–	29.1 ± 8.2	–	27 ± 7.3
	Left hand	–	32.3 ± 11.2	–	28.2 ± 6.2	–	26 ± 6.7	–	22.6 ± 7.1


*This table shows the laser-pain rating and laser evoked potential parameters (latencies and amplitudes) following real (1 Hz) and sham rTMS.*

**FIGURE 2:**
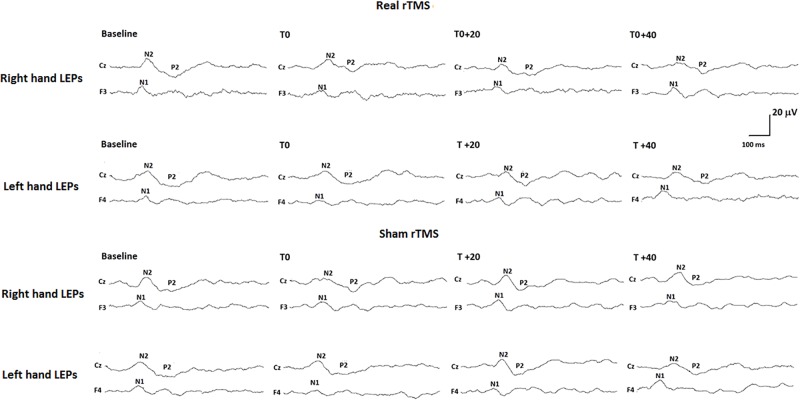
Neurophysiological findings in one representative subject. LEPs recorded to both right (controlateral to rTMS) and left (ipsilateral to rTMS) hand stimulation in the real rTMS session and to right and left hand stimulation in the sham rTMS session are shown.

Analysis of variance did not show any modification of both laser-pain rating and LEP parameters (latencies and amplitudes) to right and left hand stimulation following 1 Hz rTMS (Figure 2 and Table 2).

**Table 2:** Statistical results.

		Session		Time		Interaction (session × time)	
		*F*	*P*	*F*	*P*	*F*	*P*
VAS values	Right hand	1.87	0.18	0.72	0.54	0.12	0.95
	Left hand	0.36	0.55	1.01	0.40	0.15	0.93
LEP latencies							
N1	Right hand	0.53	0.47	0.25	0.86	0.59	0.63
	Left hand	0.05	0.82	0.18	0.91	0.36	0.78
N2	Right hand	0.13	0.72	0.20	0.89	0.05	0.99
	Left hand	0.04	0.84	0.32	0.81	0.07	0.97
P2	Right hand	0.14	0.71	2.02	0.12	0.30	0.83
	Left hand	0.48	0.49	0.59	0.62	0.72	0.54
LEP amplitudes							
N1	Right hand	2.36	0.13	0.55	0.65	0.72	0.54
	Left hand	2.06	0.16	1.34	0.27	1.62	0.20
N2/P2	Right hand	0.08	0.77	1.82	0.16	0.09	0.97
	Left hand	0.27	0.61	2.05	0.12	0.57	0.64


*This table shows statistical results of the studied sample. VAS values and LEP latencies and amplitudes underwent two-way ANOVAs, by considering the session and the time (baseline, T0, T0+20, and T0+40) as variables.*

## Discussion

In the present study, we investigated the effect of 1 Hz rTMS stimulation over contralateral M1 on LEP amplitudes and laser-pain rating. Our results showed that 1 Hz rTMS of the left M1 did not modify the laser-pain rating and the LEP amplitude to stimulation of both the contralateral (right) and ipsilateral (left) hand. As expected, also the sham rTMS did not produce any modification of both neurophysiological and psychophysiological parameters. Our subjects were all right handed and we stimulated the left M1 as the unilateral stimulation of that area is able to induce bilateral analgesic effect as previous discussed.

Previous studies suggested that high-frequency rTMS of the M1 area can reduce pain in both healthy subjects (Houzé et al., 2013; Ciampi de Andrade et al., 2014) and patients with neuropathic pain (Lefaucheur et al., 2001, 2006; Hosomi et al., 2013). The effect of high-frequency rTMS of the M1 area on the pain-related brain responses is far less clear. Indeed, while Lefaucheur et al. (2010) showed a reduction of the LEP amplitudes after stimulation of the painful hand in patients with neuropathic pain, a N2/P2 LEP amplitude reduction was described after either real or sham 10 Hz rTMS of the M1 area in healthy subjects (Bradley et al., 2016) and migraine patients (de Tommaso et al., 2010).

As for low-frequency rTMS, Tamura et al. (2004a) found that 1 Hz rTMS over M1 area increased the LEP amplitude and the laser-pain perception in healthy subjects. These results confirmed the facilitatory effect of 1 Hz rTMS over the M1 area on capsaicin-induced pain (Tamura et al., 2004b). The repetition of 3 low-frequency rTMS sessions, at the interval of 2 weeks from each other, produced inconsistent results in patients affected by neuropathic pain (André-Obadia et al., 2006). In a more recent study, 1 Hz rTMS of the M1 area did not have any effect on heat and cold pain thresholds, while it increased both pain thresholds if primed by cathodal transcranial direct current stimulation (tDCS) and decreased them if primed by anodal tDCS (Moloney and Witney, 2013). Those results could be explained by the concept of meta-plasticity induced effect that is the capability of tDCS of reversing the effect of rTMS. This phenomena acts both for motor (Siebner et al., 2004) and visual cortex (Bocci et al., 2014). tDCS can be also used to directly stimulate cerebral areas in order to obtain an analgesic effect (Moisset and Lefaucheur, 2018). tDCS has been applied also over the cerebellum to modify pain perception (Bocci et al., 2015) as the cerebellum proved to have modulatory effect over M1 area (Ates et al., 2018).

The putative effect of rTMS of the M1 area on pain could depend on two elements: (1) the modification of M1 excitability after either low- or high-frequency stimulation and (2) the tonic effect of M1 activity on the pain matrix. While high-frequency rTMS is thought to increase the M1 excitability (Pascual-Leone et al., 1998; Lefaucheur et al., 2008), low-frequency rTMS showed an inhibitory effect (Maeda et al., 2000; Touge et al., 2001; Phielipp et al., 2017). As for the influence of M1 area on pain, experimental studies suggested that the M1 activation, which occurs during movement or preparation to it, has an analgesic effect (Kakigi and Shibasaki, 1992; Kakigi et al., 1993; Nakata et al., 2004; Le Pera et al., 2007). From this point of view, while increasing the M1 area activity should lead to pain reduction, its inhibition should have a pro-algesic effect. Therefore, one could argue that while high-frequency rTMS should produce analgesia by increasing the M1 excitability, low-frequency rTMS should facilitate any nociceptive activity by inhibiting the motor cortex. Although this assumption has not always been confirmed by both clinical and experimental studies (see above), the therapeutic use of high-frequency rTMS in pain condition is supported by evidence (Lefaucheur et al., 2014). The results of Tamura et al. (2004a) agree with the hypothesis of a tonic inhibitory action of M1 area on pain. Indeed, M1 inhibition driven by 1 Hz rTMS dampened the N2-P2 LEP amplitude and the subjective perception of laser-pain. On the contrary, we failed in demonstrating any modification on both pain perception and pain-related brain responses following 1 Hz rTMS over the M1 area. The disagreement between our results and those by Tamura et al. can be due to different elements. First, while in our study rTMS consisted of a total of 1200 stimuli in 20’, Tamura et al. delivered 600 stimuli in 10 min. We cannot exclude that a possible 1 Hz rTMS pro-algesic effect occurs in the first part of stimulation and can get lost with prolonging the treatment. Second, a possible placebo/nocebo effect is also to be considered. Indeed, it is accepted that the expectation to feel more or less pain can influence pain perception (Colloca et al., 2004; Benedetti et al., 2007; Bradley et al., 2016). This effect can influence the outcome of a drug therapy as well as a non-invasive stimulation. Not only the way in which the information is given but also the words that are used can prepare subject to expect something. For that reason in our protocol we did not mention the words “pain” or “analgesia,” as we demonstrated that the mere verbal suggestion in healthy subject was able to produce either placebo or nocebo effects (Colloca et al., 2008; Pazzaglia et al., 2016). Seen in this light, Tamura’ results could have been influenced by nocebo mechanisms which possibly affected laser-pain rating and LEP amplitude more than rTMS.

### Limitations of the Study

Some limitations of the current study have to be pointed out. First, the small number of the enrolled subjects could have affected the final results. In spite of this, the cross over design of the study, where every subject was control of him/herself, lead to homogenous findings.

Second, subtle differences in LEPs induced by rTMS may have been missed due to the use of a rough LEP recording technique. Indeed, neither we used high density EEG recording, nor we performed updated techniques of brain signal analysis, such as dipole modeling or coherence investigation. Although we cannot exclude that the use of more refined methods of recording and/or analysis could lead to some positive results, we must underline that the present negative neurophysiological findings parallel the lack of any psychophysical change of pain rating after rTMS.

Third, we studied healthy subjects, thus any immediate translation of the present results to patients affected by chronic pain conditions is not allowed. Indeed, there are several differences between healthy subjects and patients, since the basal status of cortical excitability is modified by long-lasting pain.

## Conclusion

Our study failed in showing LEP and laser-pain modification induced by 1 Hz rTMS of the primary motor cortex. This, however, cannot be taken as an evidence against either the inhibitory action of M1 area on pain or that of rTMS on the motor cortex. Indeed, we must consider that pain produced by laser pulses is phasic and deeply different from clinical pain. Therefore, we cannot exclude that 1 Hz rTMS over the M1 area can change the perception of a more ecological tonic pain and its neurophysiological correlate. Nevertheless, our study suggests that the functional network connecting the motor cortex with the pain matrix areas is complex and cannot be trivialized to mere reciprocal inhibitory/facilitatory actions. The environmental context is probably very important and determines the results which can be obtained in different experimental and clinical situations.

## Author Contributions

CP and CV performed the literature survey. ET, DC, and GG performed the patients’ recruitment and data collection. CP, LP, and MV analyzed the data. CP, CV, ET, DC, and MV wrote the manuscript. CP, LP, and MV cooperated in research management and supervising the research project. All authors contributed to the experimental design, reviewed the manuscript, and approved the final version.

## Conflict of Interest Statement

The authors declare that the research was conducted in the absence of any commercial or financial relationships that could be construed as a potential conflict of interest.
